# Knowledge and Practice of Contact Lens Users in Eastern Province, Saudi Arabia

**DOI:** 10.7759/cureus.72091

**Published:** 2024-10-22

**Authors:** Maryam A Al Najjar, Alhwraa S Almajed, Ryan J AlGhanem, Omaima M AlYahya, Hajar M AlHajri, Abdullah Almaqhawi

**Affiliations:** 1 Family and Community Medicine, King Faisal University, Al Ahsa, SAU

**Keywords:** colored cosmetic contact lens, corrective contact lens, eyeglasses, females, knowledge, practice

## Abstract

Introduction

Contact lenses (CLs) are temporary prosthetic devices designed to fit directly on the cornea and are commonly used to correct refractive errors. Since CLs are easily accessible, the users might lack the basic knowledge of the safety and complications of such products. Investigating the patterns and behaviors related to using CLs can identify the factors that potentially play a role in developing complications.

Aim

This study aimed to evaluate the awareness and utilization of lens care products among CL wearers.

Subject and methods

This observational cross-sectional study was conducted among adult women in the Eastern Province of Saudi Arabia. A self-administered questionnaire was distributed among women using an online survey. The questionnaire includes demographic characteristics, knowledge, practices, reasons, causes, and side effects of CLs.

Results

Of the 393 women, 68.4% were aged between 18 and 30 years. The most common source of CL information was a social circle (27%). Interestingly, parties and occasions were the most influencing factors (83.7%), while dryness of the eyes was the most common side effect (52.2%). According to the result, the rate of regular CL wearers was 16% and significant predictors of regular CL wearers were wearing CL due to medical and cosmetic reasons.

Conclusion

The use of CLs was common in women living in the Eastern Province. Independent predictors of regular CL use were eyeglass use, wearing of CL for cosmetic and medical purposes, and sleeping with CL, while dryness of the eye could be the most critical risk factor for daily use of CL. Prospective studies in nature are needed to determine its cause and effect.

## Introduction

Contact lenses (CL) are temporary prosthetic devices that are designed to fit directly on the cornea and are commonly used for the correction of refractive errors [1]. Around the world, more than 140 million people of different age groups wear CLs for different purposes, such as optical, therapeutic, or cosmetic causes [2]. The cornea can undergo many substantial changes as a result of the use of CLs, and these changes vary depending on the type of lens being worn (i.e., different CL materials and wearing habits) [3]. A study that was done in Saudi Arabia showed that 51.4% of people who use CL had underlying eye problems. Many people with refractive error found that CLs improved their quality of life compared to wearing eyeglasses [4].

Due to the fact that CLs are easily accessible, the users might lack the basic knowledge of the safety and complications of such products [4]. Improper actions of CL use, such as sleeping with the CL and poor hand and lens case hygiene, can precipitate the development of noninfectious and infectious complications such as eye dryness, keratitis, corneal inflammation, and allergic reactions of the cornea [5]. These complications can range in severity from mild to sight-threatening. It is apparent that 86% of the survey participants, which was collected in Saudi Arabia, responded that the most frequent unsafe practice was sleeping/napping with the CLs [4]. Unfortunately, a study that was conducted in the United States concluded that 32.9% of CL users reported that they had never been informed about any advice or guidelines regarding the use of CL [6].

Data from several studies have identified that microbial keratitis is one of the most common and serious complications of CL [7,8]. To illustrate, a previous study that was conducted in the United States revealed that about one million cases of keratitis clinical visits were related to CL complications with a cost that reached up to 175$ million [9]. It has been shown that prescribing CL from an authorized place is crucial to learning the proper usage and eye care routine, as it has been concluded that many of the Saudi Arabian population did not seek eye examination prior to the utilization of CL [10].

Investigating the patterns and behaviors related to the use of CLs can identify the factors that potentially play a role in the development of complications. A comprehensive study of CL use aids in improving eye care and health and guiding the development of interventions aiming to minimize possible risks and enhance the safe use of CLs. Because multiple studies concluded that the Saudi Arabian population lacks knowledge about the safe use of CL, our aim of this study is to determine the individual’s knowledge and practice for CL users and to assess their knowledge of some complications regarding improper CL use in Eastern Province, Saudi Arabia.

## Materials and methods

This is an observational cross-sectional study that was conducted in Eastern Province, Saudi Arabia, using an online questionnaire (see Appendix). The data were collected from adult females (aged 18 and above) in Eastern Province, Saudi Arabia. The sample size was selected using a convenient random sampling technique, which was done by distributing the questionnaire on social media in different applications. The questionnaire builds upon previous research conducted in Riyadh by Othman Alzahrani and other authors titled “Contact Lens Practices and Knowledge of Complications and its Association With Refractive Error in Saudi Arabia.” By applying the formula n = z^2^pq/d^2^, the sample size was determined. A 50% estimated proportion, a 95% level of confidence, and a 5% degree of precision. A minimum sample size of 385 was determined. To guarantee the sufficiency and accuracy of the results, more participants and candidates were included. Inclusion criteria include females aged 18 and above from all nationalities who are residents of the Eastern Province of Saudi Arabia and have consented to participate in the study. Exclusion criteria exclude males and females younger than 18 years old, non-residents in Eastern Province, Saudi Arabia, and those not consenting to participate in the study. Institutional Review Board and Ethics Committee Information was taken from the Institutional Review Board of King Faisal University with the following approval number: KFU-REC-2024-JA-ETHICS1956.

Statistical analysis

The data were analyzed using the software program Statistical Packages for Software Sciences (SPSS) (IBM SPSS Statistics for Windows, IBM Corp., Version 26, Armonk, NY). Descriptive statistics were given as numbers and percentages (%) for all categorical variables. The relationship between the type of CL users among the demographic and the practices-related characteristics of female CL wearers has been conducted using the chi-square test. Significant results were then tested in a multivariate regression analysis to determine the significant independent predictor of regular use of CLs with corresponding odds ratios and 95% confidence intervals. Values were considered significant with a p-value of less than 0.05.

Confidentiality and ethical consideration

The highest level of confidentiality was used while handling data. Every study participant's privacy was safeguarded at all times. For approval, the King Faisal University in Saudi Arabia's Deanship of Scientific Research's ethics committee was consulted.

## Results

This study enrolled 393 female CL wearers. As seen in Table 1, the majority were aged between 18 and 30 years old (269, 68.4%). Two hundred twenty-five (57.3%) were visiting an ophthalmologist when needed. Additionally, 70 (17.8%) were using eyeglasses for one to five years.

**Table 1:** Demographic characteristics of female contact lens wearers (n = 393)

Study variables	n (%)
Age group	
18-30 years	269 (68.4%)
31-40 years	82 (20.9%)
41-50 years	36 (09.2%)
51-60 years	06 (01.5%)
Do you visit an ophthalmologist?	
No	132 (33.6%)
Regularly	29 (07.4%)
When needed	225 (57.3%)
When you wanted to buy contact lens	07 (01.8%)
Duration of using eyeglasses	
<1 year	41 (10.4%)
1-5 years	70 (17.8%)
6-10 years	45 (11.5%)
>10 years	60 (15.3%)
Did not use eyeglasses	177 (45.0%)

When reviewing participants' answers about CLs in Table 2, it was observed that the most common cause of CL use was cosmetic 228 (58.0%). Most were part-timer CL users (243, 61.8%), and the monthly lens was the most preferred CL (327, 83.2%). Approximately 312 (79.4%) were wearing CL according to their own choice. The most common source of CL information was a social circle (106, 27.0%).

**Table 2:** Knowledge of CLs used (n = 393) CL, contact lens ^†^ Variable with multiple response answers.

Study variables	n (%)
The cause of CL use	
Medical	139 (35.4%)
Cosmetic	228 (58.0%)
Both (medical and cosmetic)	26 (06.6%)
The duration of CL use	
All the time	63 (16.0%)
Part-time	243 (61.8%)
I used to wear CLs in the past	87 (22.1%)
Type of CL used ^†^	
One day lens	76 (19.3%)
Weekly lens	20 (05.1%)
Monthly lens	327 (83.2%)
How did the participants start using CLs?	
I started on my own choice	312 (79.4%)
I started based on a doctor's recommendation	11 (02.8%)
I started based on advice from others	19 (04.8%)
I started to be like others	24 (06.1%)
Others	27 (06.9%)
Source of CL information	
Ophthalmologist	103 (26.2%)
Social circle	106 (27.0%)
No such information was given	119 (30.3%)
Social media (Instagram, Snapchat)	65 (16.5%)

**Figure 1:**
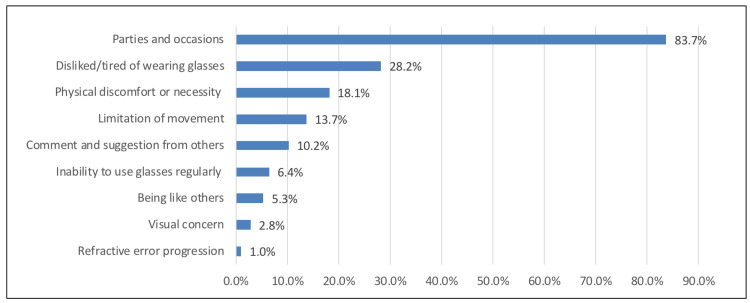
Reasons influencing the decision to wear contact lenses

Figure 1 illustrates the most common reasons influencing the decision to wear CLs. According to multiple response answers, the most common reasons were parties and occasions (329, 83.7%), followed by dislike/tiredness of wearing glasses (111, 28.2%), and physical discomfort or necessity (71, 18.1%).

**Figure 2:**
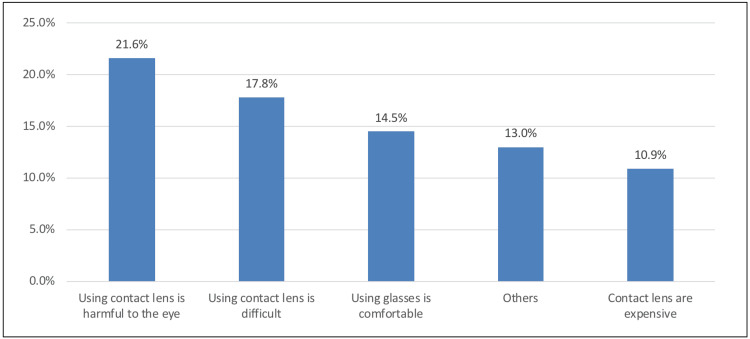
Causes to avoid wearing contact lenses

Figure 2 showed that the most common barrier to wearing CL was that the use of CL is harmful to the eye (85, 21.6%), followed by that CL is difficult to wear (70, 17.8%) and eyeglasses being more comfortable (57, 14.5%).

**Figure 3:**
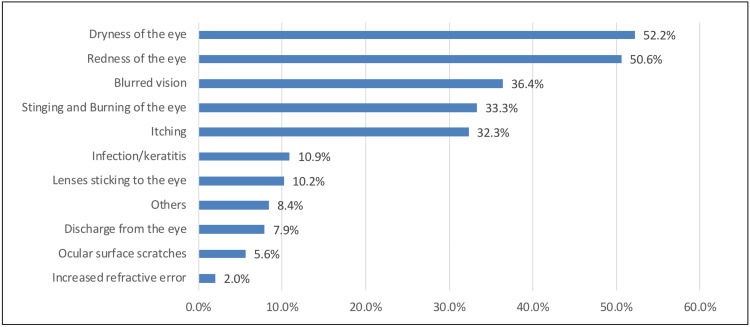
Adverse effects of wearing contact lenses

In Figure 3, the common side effect of wearing CLs was dryness of the eye (205, 52.2%), followed by redness of the eye (199, 50.6%) and blurred vision (143, 36.4%).

**Figure 4:**
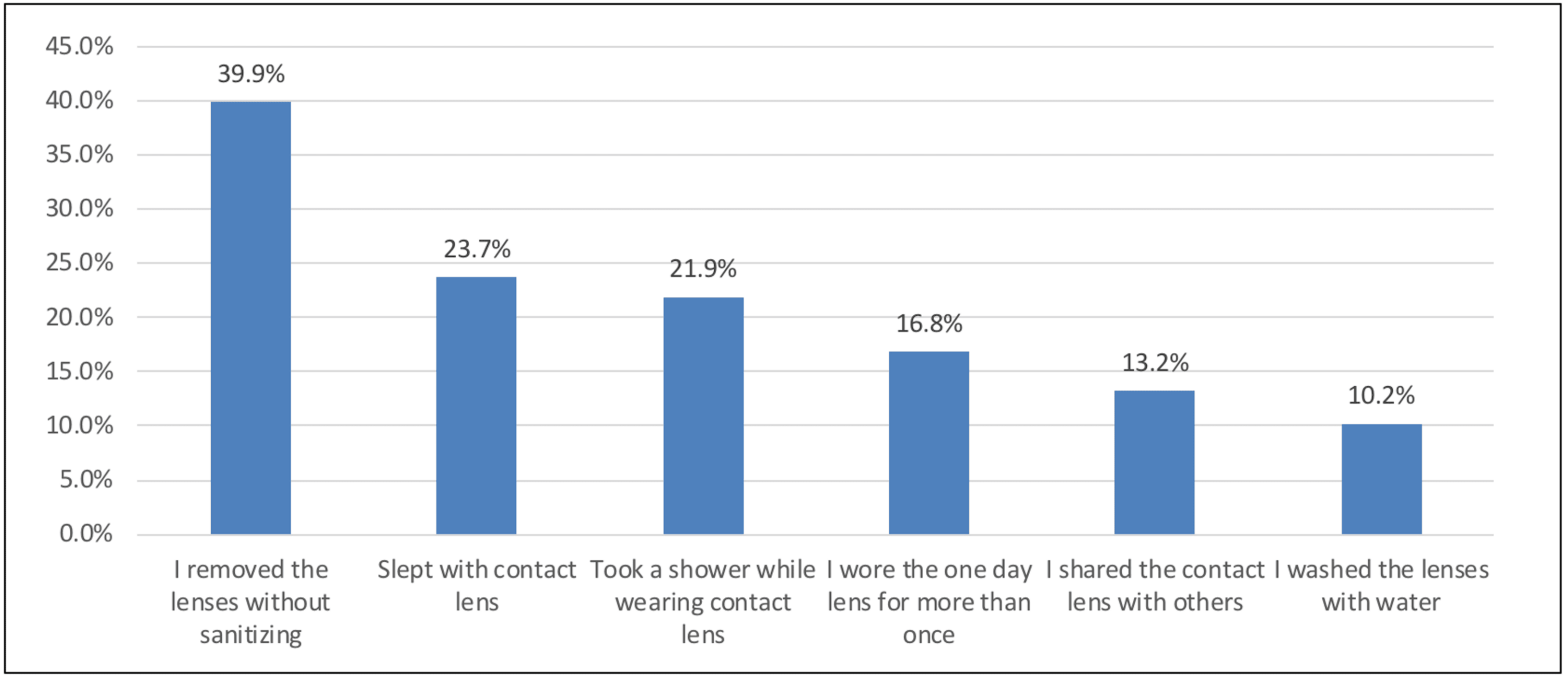
Most common malpractices when wearing contact lenses

In Figure 4, the most common malpractices of females when wearing CLs were removing the lenses without sanitizing (157, 39.9%), sleeping with CL (93, 23.7%), and taking a shower while wearing CL (86, 21.9%).

In Table 3, results revealed that regular wearers of CL were more likely females who visited an ophthalmologist (p < 0.001), wore eyeglasses (p < 0.001), wore CLs for medical reasons (p < 0.001), received CLs information from an ophthalmologist (p = 0.009). Regular wearers of CL experienced side effects such as infection/keratitis (p = 0.002), itching (p = 0.025), lenses sticking to the eye (p = 0.037), ocular surface scratches (p = 0.007), discharge from the eye (p = 0.040) and dryness of the eye (p < 0.001). The malpractices of regular CL wearers are taking a shower while wearing CL (p < 0.001), sleeping with CL (p < 0.001), wearing one-day lens more than once (p < 0.001), washing the lenses with water (p = 0.019) and not changing the CL solution (p < 0.001). In contrast, females who share their CL with others are more likely to be non-regular CL users (p = 0.001).

**Table 3:** Relationship between the type of CL user among demographic characteristics and the practices of females on wearing CLs (n = 393) CL, contact lens ^†^ Variable with multiple response answers. ^§^ p-value has been calculated using the chi-square test. ^**^ Significant at p < 0.05 level.

Factor	Type of CL user		p-value ^§^
	Regular n (%)^(n=63)^	Non-regular n (%) ^(n=330)^	
Age group			
≤30 years	48 (76.2%)	221 (67.0%)	0.149
>30 years	15 (23.8%)	109 (33.0%)	
Visited an ophthalmologist			
Yes	53 (84.1%)	208 (63.0%)	<0.001 ^**^
No	10 (15.9%)	122 (37.0%)	
Use of eyeglasses			
Yes	50 (79.4%)	166 (50.3%)	<0.001 ^**^
No	13 (20.6%)	164 (49.7%)	
The cause of CL use			
Medical	52 (82.5%)	87 (26.4%)	<0.001 ^**^
Cosmetic	04 (06.3%)	224 (67.9%)	
Both (medical and cosmetic)	07 (11.1%)	19 (05.8%)	
How did the participants start using CLs?			
Own decision	52 (82.5%)	260 (78.8%)	0.500
Recommended by doctor/others	11 (17.5%)	70 (21.2%)	
Source of CL information			
Ophthalmologist	27 (42.9%)	76 (23.0%)	0.009 ^**^
Social circle	15 (23.8%)	91 (27.6%)	
No such information was given	15 (23.8%)	104 (31.5%)	
Social media (Instagram, Snapchat)	06 (09.5%)	59 (17.9%)	
Adverse effect of wearing CLs ^†^			
Increased refractive error	0	08 (02.4%)	0.365
Infection/keratitis	14 (22.2%)	29 (08.8%)	0.002 ^**^
Itching	28 (44.4%)	99 (30.0%)	0.025 ^**^
Lenses sticking to the eye	11 (17.5%)	29 (08.8%)	0.037 ^**^
Stinging and burning of the eye	21 (33.3%)	110 (33.3%)	1.000
Redness of the eye	29 (46.0%)	170 (51.5%)	0.425
Blurred vision	20 (31.7%)	123 (37.3%)	0.403
Ocular surface scratches	08 (12.7%)	14 (04.2%)	0.007 ^**^
Discharge from the eye	09 (14.3%)	22 (06.7%)	0.040 ^**^
Dryness of the eye	52 (82.5%)	153 (46.4%)	<0.001 ^**^
Most common malpractices when wearing CLs ^†^			
Took a shower while wearing CL	38 (60.3%)	48 (14.5%)	<0.001 ^**^
Slept with CL	39 (61.9%)	54 (16.4%)	<0.001 ^**^
I wore the one-day lens for more than once	23 (36.5%)	43 (13.0%)	<0.001 ^**^
I shared the CL with others	0	52 (15.8%)	0.001 ^**^
I washed the lenses with water	24 (38.1%)	79 (23.9%)	0.019 ^**^
I removed the lenses without sanitizing	31 (49.2%)	126 (38.2%)	0.102
I did not change the CL solution	29 (46.0%)	79 (23.9%)	<0.001 ^**^

When conducting a multivariate regression analysis (Table 4), it was revealed that compared to females who do not wear eyeglasses, females who wear eyeglasses were 12.9 times more likely to be regular CL wearers (adjusted odds ratio (AOR) = 12.941; CI = 2.948-56.812; p = 0.001). Compared to females who used CLs for medical reasons, females who used CLs for both medical and cosmetic reasons had an increased chance of being regular CL wearers by at least 84.9 times higher (AOR = 84.951; 95% CI = 11.12-649.2; p < 0.001). Incidentally, females who were regular CL wearers were at an increased risk of dryness of the eyes by at least 2.53 times higher (AOR = 1.035; 95% CI = 1.035-6.168; p = 0.042). In addition, females who slept with CLs were 2.91 times more likely to be regular CL wearers (AOR = 2.909; 95% CI = 1.064-7.957; p = 0.038).

**Table 4:** Multivariate regression analysis to determine the significant independent predictor of regular use of CLs (n = 393) AOR, adjusted odds ratio; CL, contact lens ^†^ Variable with multiple response answers. ^§^ P-value has been calculated using the chi-square test. ^**^ Significant at p<0.05 level.

Factor	AOR	95% CI	p-value
Visited an ophthalmologist			
Yes	2.194	0.767-6.270	0.143
No	Ref	-	-
Use of eyeglasses			
Yes	12.941	2.948-56.812	0.001 **
No	Ref	-	-
The cause of CL use			
Medical	Ref	-	-
Cosmetic	0.517	0.169-1.585	0.248
Both (medical and cosmetic)	84.951	11.12-649.2	<0.001 **
Source of CL information			
Ophthalmologist	Ref	-	-
Social circle	1.021	0.268-3.892	0.975
No such information was given	0.700	0.170-2.875	0.621
Social media (Instagram, Snapchat)	0.905	0.221-3.712	0.889
Adverse effect of wearing CLs ^†^			
Infection/keratitis	1.524	0.447-5.190	0.501
Itching	0.733	0.335-1.601	0.436
Lenses sticking to the eye	0.413	0.129-1.320	0.136
Ocular surface scratches	1.422	0.303-6.680	0.656
Discharge from the eye	2.026	0.580-7.082	0.269
Dryness of the eye	2.526	1.035-6.168	0.042 ^**^
Most common malpractices when wearing CLs ^†^			
Took a shower while wearing CL	1.989	0.748-5.284	0.168
Slept with CL	2.909	1.064-7.957	0.038 ^**^
I wore the one-day lens for more than once	1.568	0.666-3.694	0.303
I washed the lenses with water	2.166	0.872-5.385	0.096
I did not change the CL solution	1.497	0.645-3.474	0.348

## Discussion

Prevalence of CL wearers

The present study evaluated the knowledge and practice of CL users among women living in the Eastern Province of Saudi Arabia. According to our results, the prevalence of regular CL wearers was 16%. This is consistent with the study done in Malaysia [5], which had a prevalence of 9.9%. Supporting these reports, a study conducted in Kenya [11] documented a very low prevalence and knowledge of CL wearers, which was concurred by the paper of Timothy et al. (2023) [12]. Contradicting these reports, Teo et al. (2011) [10] reported a high prevalence of daily CL wearers at 85.2%. Appropriate understanding and practice of CL are necessary among regular CL wearers. Many utilize CLs without prescription and exclusively for cosmetic purposes only. Therefore, continuous efforts are needed to educate users about the improper use of CLs to decrease the incidence of CLs-related complications.

Significant factors of regular wearing of CL

Data from this study suggest that regular CL wearers tended to visit an ophthalmologist, use eyeglasses, use CL due to eye problems, and have a source of CL information from an ophthalmologist. Moreover, regular CL wearers’ common malpractices are taking a shower while wearing CL, sleeping with CL, wearing one-day lens more than once, washing the lenses with water, and not changing the CL solution. In addition, regular CL wearers tend to experience CL-related complications such as infection/keratitis, itching, lenses sticking to the eye, stinging and burning of the eye, ocular surface scratches, discharge from the eye, and dryness of the eye. However, in our multivariate regression model, only using eyeglasses, wearing CL due to medical and cosmetic reasons, experiencing dryness of the eye, and sleeping with CL remained significant and determined as the independent predictors of CL's regular use (p < 0.05). This is almost consistent with the previous reports done by Alzahrani et al. (2021) [4]. In Thailand [13], poor CL behavior was dependent mainly on purchasing lenses from the internet, long wear duration, and wearing CL for more than one year. However, in Riyadh [14], poor knowledge was significantly associated with occasional users and those without CL prescription; however, knowledge did not differ significantly by gender, education, and type of work (p > 0.05).

Sources of CL information

Source of information plays a vital role in learning the basic facts about CL. In our study, social circles (27%), an ophthalmologist (26.2%), and social media (16.5%) were the most prominent sources of CL information. This mirrored the results of Alzahrani et al. (2021) [4], wherein social circle was also CL wearers' most well-known source of information. Moreover, in India, first-time CL wearers indicated friends and ophthalmologists were the most common sources of CL information. Regarding the social circle, although 26% surely recommended CL to others in the future, most CL wearers did not suggest the use of CL to non-CL wearers [15].

Influential factors to wear CL

Women in this study were influenced mainly by parties and occasions to wear CLs (83.7%). Other influencing factors were less rated, including tiredness of wearing glasses (28.2%), physical discomfort (18.1%), limitation of movement (13.7%), suggestion from others (10.2%), not being able to wear glasses regularly (6.4%), mimicking like others (5.3%), visual concern (2.8%), and refractive error as the least influencing factor (2%). In Riyadh [4], mimicking others was the most influential reason to wear CL, while in Malaysia [5], cosmesis (42.8%) and comfort (32.7%) were the primary reasons among secondary school students.

Causes to avoid wearing CL

More than one-fifth of the respondents (21.6%) cited "CL is harmful to the eye" as the most common reason to avoid CL, followed by "wearing CL is difficult" (17.8%) and "using glasses is more comfortable" (14.5%), while "CLs are expensive" being the least reason to avoid CL (10.9%). It can be assumed that respondents were more interested in wearing CLs, regardless of any negative effects of wearing them.

Side effects of CL

Incidentally, many of the respondents experienced side effects while wearing CL, and the most common of them were dryness of the eye (52.2%), redness of the eye (50.6%), blurred vision (36.4%), stinging and burning of the eye (33.3%), and itching (32.3%). Others also experienced discharge from the eye (7.9%), ocular surface scratches (5.6%), and increased refractive error (2%). Consistent with these results, Cope et al. (2015) [2] found that nearly all (99%) CL wearers showed at least single-related risk complications, with red or painful eyes being the most common. However, in a study by Teo et al. (2022) [10], one out of four CL wearers (25.6%) were diagnosed with infected keratitis, with some of them (55 patients) being hospitalized to manage the infection. Soft disposable CL was identified as the most common reason for infective keratitis (73%).

Most common malpractices when wearing CL

The practices toward wearing CLs seem to be lacking. Nearly 40% of the respondents removed the lenses without sanitizing them; 23.7% and 21.9% slept or took a shower while wearing CL. Others wore the one-day lens multiple times (16.8%) or shared their CL with others (13.2%) and even washed the lens with water (10.2%). These practices may have been consistent with the students in India [16]. Accordingly, they noted that not removing CL before bed was the practice of more than half of the students (55%); 58% were not aware of the duration of cleaning CL by solution, while 28% were still using CL beyond expiry dates. In contrast, students in Malaysia [17,18] demonstrated better practices in wearing CL. Most of them (88.9%) followed hand hygiene before wearing CL, and 85.7% asserted that they adhere strictly to sanitizing CL. To conclude, compliance with proper CL use is necessary to prevent CL-related complications.

Limitations

The cross-sectional design of this study and the use of an online, widely distributed questionnaire to gather data may have affected the accuracy of the findings. Nevertheless, this survey offers CL wearers a helpful starting point for understanding their awareness of lens care products in the Saudi Arabian Eastern Province. Furthermore, the bias-free status of Knowledge and Practice of Contact Lens Users would be more properly reflected by a larger sample size from a population-based survey.

## Conclusions

Sixteen percent of women in the Eastern Province were regular CL wearers. The use of eyeglasses and the use of CL for medical and cosmetic reasons were identified as significant factors in CLs’ regular use. Interestingly, parties and occasions were the most prominent reasons for wearing CLs, while the harmful effect on the eye was the main reason for avoiding it. There were gaps in the knowledge and practice of using CLs. Hence, health communication policies and continuous public ocular health education that can stimulate behavior changes among CL users are needed to bridge the gaps. Health education should focus on those using CLs for cosmetic reasons and purchasing without prescription to enhance their CL-related practice and decrease eye-related infections.

## Appendices

**Table 5:** Questionnaire CL, contact lens

Knowledge and practice of CL users in Eastern Province, Saudi Arabia
Gender:
Male
Female
Age group:
18-30
31-40
41-50
51-60
Above 60
Educational level:
Primary school
High school
University
Income level:
Less than 5,000 Saudi Riyals
5,000-15,000 Saudi Riyals
Above 15,000 Saudi Riyals
Duration of using glasses:
Less than 1 year
1-5 years
5-10 years
More than 10 years
No use of glasses
CL use:
Persistent use
Intermittent use
Previously used
Never used
Visits ophthalmologist:
Regularly
When needed
When wanting to buy CL
Others
Levels of importance of participants’ reasons for CL avoidance. List upon importance:
Using CLs is difficult
Using glasses is comfortable
Using CLs is harmful to the eye
My ophthalmologist does not recommend CLs
CLs are expensive
Concerns related to CL use among participants who did not use CLs
Problem with near vision
Increased refractive error
Infection
Itching
Lenses sticking to the eye
Lenses decentration
Stinging
Burning
Redness
Blurred vision
Ocular surface scratches
Discharge
Evening discomfort
Dryness
Cataract development
Prevent future laser surgery
Eye loss
List upon importance:
Personal observation
Immediate social circle
Information from ophthalmologist
Print/visual media
No concerned
Others
How the participants started using CLs. Choose one:
I started on my own volition
I started based on doctor recommendation
I started based on advice from others
I started to emulate others
Others
Importance of reasons influencing the decision to start using CLs. List upon importance:
Disliked/tired of wearing glasses
Physical discomfort (weight on face, headache, etc.) or necessity (facial structure, eye structure, etc.)
Esthetic/visual concern
Comments and suggestions from others
Emulation of others
Disliked/tired of wearing glasses
Inability to use glasses regularly (difficulties forgetting, carrying, etc.)
Refractive error progression
Limitation of movement (wearing glasses inconvenient while working/professional reasons, sport, etc.)
Frequency of CL-related problems experienced by CL users:
Difficulty with near vision
Increased refractive error
Infection/keratitis
Itching
Lenses sticking to the eye
Lenses decentration
Stinging
Burning
Redness
Blurred vision
Ocular surface scratches
Discharge
Evening discomfort
Dryness
Source of information about CL use. Choose one:
Ophthalmologist
Optician
Social circle
No such information was given
Others
Ophthalmologist and Optician
Types of CL worn:
Soft
Rigid
Gas permeable
Orthokeratology
Others
Duration since first CL. Using time:
All time
Part-time
Not using now
Compliance with safety measures (risk factor/behavior). Recommendation for the recent visit (yes, no):
Avoid sleeping overnight or napping in lenses
Wash and dry hands before inserting or removing lenses
Replace lenses as often as recommended
Replace lens case at least every three months
Avoid storing lenses in water
Avoid rinsing lenses in water
Avoid “topping off” solution
Avoid swimming in lenses
Avoid showering in lenses
Avoid sharing lenses
Avoid sharing lenses
Did visit eye doctor at least annually
Heard or stated none of recommendations
